# Crystal structure of 4,5-di­bromo­phenanthrene

**DOI:** 10.1107/S2056989017003863

**Published:** 2017-03-21

**Authors:** Nicholas S. Kim, Dasan M. Thamattoor

**Affiliations:** a Department of Chemistry, Colby College, Waterville, ME 04901, USA

**Keywords:** crystal structure, polycyclic aromatic hydro­carbon, helical mol­ecule

## Abstract

The synthesis and crystal structure of the title compound, C_14_H_8_Br_2_, is described. The mol­ecule is positioned on a twofold rotation axis and the asymmetric unit consists of half a mol­ecule with the other half being generated by symmetry. The presence of two large bromine atoms in the bay region significantly distorts the mol­ecule from planarity and the mean planes of the two terminal rings of the phenanthrene system are twisted away from each other by 28.51 (14)°. The torsion angle between the two C—Br bonds is 74.70 (14)° and the distance between the two Br atoms is 3.2777 (13) Å. The mol­ecules pack in layers in the crystal, with the centroids of the central rings of the phenanthrene units in adjacent layers separated by a distance of 4.0287 (10) Å. These centroids are shifted by 2.266 (6) Å relative to each other, indicating slippage in the stacking arrangement. Furthermore, the distance between the centroids of the terminal and central rings of the phenanthrene units in adjacent layers is slightly shorter at 3.7533 (19) Å. While all of the mol­ecules within each layer are oriented in the same direction, those in adjacent layers are oriented in the opposite direction, leading to anti-parallel stacks.

## Abstract 2

The mol­ecule is positioned on a twofold rotation axis and the asymmetric unit consists of half a mol­ecule with the other half being generated by symmetry. The presence of two large bromine atoms in the bay region significantly distorts the mol­ecule from planarity. The mol­ecules pack in layers in the crystal with slippage in the stacking arrangement. While all of the mol­ecules within each layer are oriented in the same direction, those in adjacent layers are oriented in the opposite direction, leading to anti-parallel stacks.

## Chemical context   

**Figure 1:**
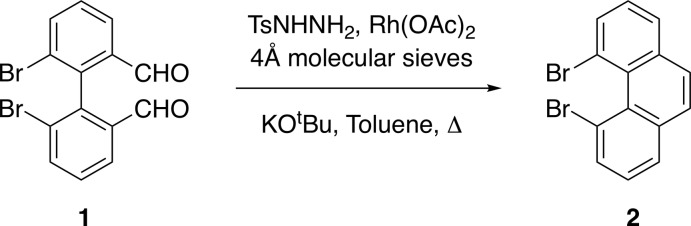
Synthesis of 4,5-di­bromo­phenanthrene (**2**).

In the course of our research into non-planar polycyclic hydro­carbons, we became inter­ested in the preparation of helical phenanthrene systems bearing bulky substituents in the 4- and 5-positions. Towards that end, we undertook the synthesis of 4,5-di­bromo­phenanthrene (**2**) from the known di­aldehyde **1** (Suzuki *et al.*, 2009 ▸) using a recently published procedure (Xia *et al.*, 2012 ▸), as shown in Fig. 1 ▸. Although there is one reference to the title compound **2** in the literature (Cosmo *et al.*, 1987*a* ▸), neither the procedure for its synthesis nor its X-ray crystal structure has previously been reported.
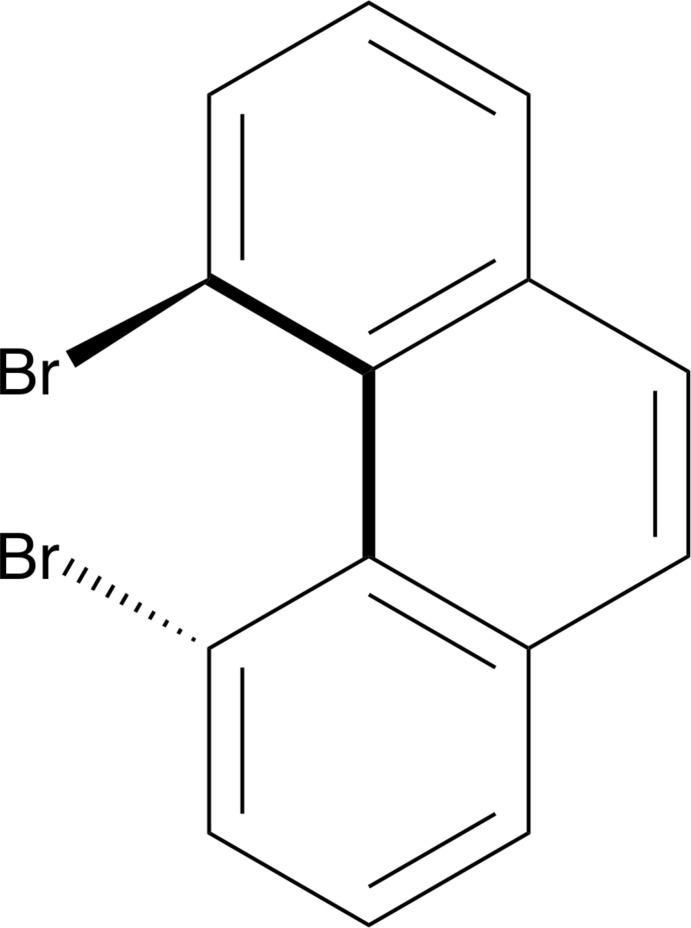


## Structural commentary   

**Figure 2:**
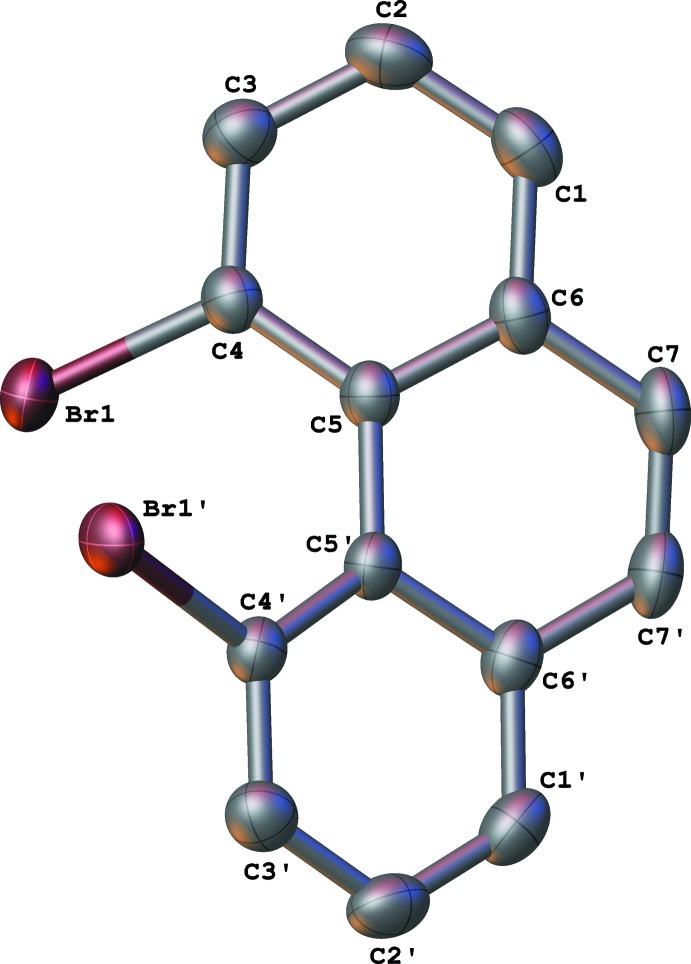
Crystal structure of **2** with displacement ellipsoids shown at the 50% probability level. H atoms omitted for clarity. [Symmetry code: (’) 1 − *x*, + *y*, 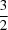 − *z*].

**Figure 3:**
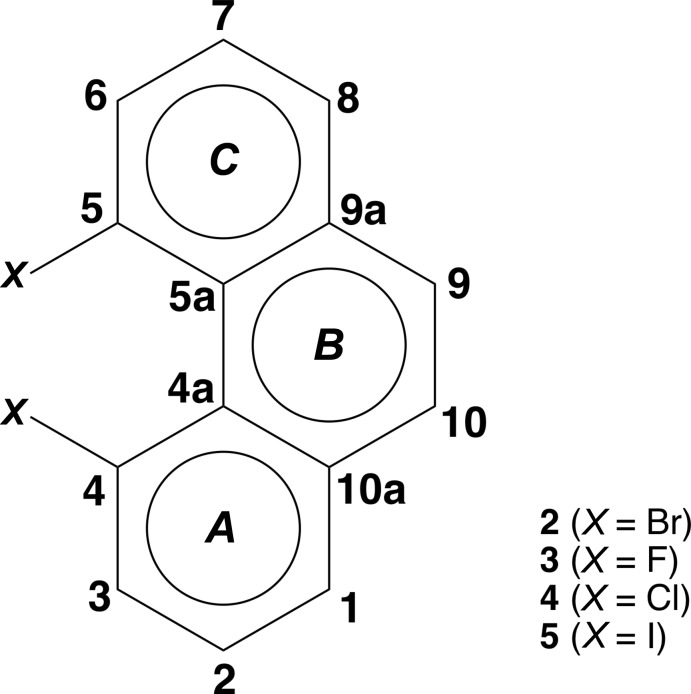
The 4,5-dihalo derivatives of phenanthrene shown with conventional chemical numbering. This figure is used as a reference for the data in Table 1 ▸.

The asymmetric unit consists of half a mol­ecule with the other half generated by symmetry as the mol­ecule is positioned on a twofold rotation axis that bis­ects the central ring. The crystal structure shows a deformed phenanthrene framework (Fig. 2 ▸) in which the planes of the two terminal rings are twisted away from each other by 28.51 (14)° and the torsion angle between the two C—Br bonds (Br1—C4—C4′—Br1′) is 74.70 (14)°. The C4—C5—C5′—C4′ torsion angle is 32.8 (6)°, and the distance between the two bromine atoms is 3.277 (13) Å, a value consistent with a previous report (Cosmo *et al.*, 1987*a* ▸). A comparison of the key structural features of the title compound **2** to those of other known 4,5-dihalo­phenanthrenes (Cosmo *et al.*, 1987*b* ▸; Bock *et al.*, 1998 ▸) is presented in Table 1 ▸ with reference to the general structure shown in Fig. 3 ▸. The distance between the two halogen atoms, and the torsion angle between the two carbon–halogen bonds (*X*—C4—C5—*X*), increase as expected with the increasing size of the halogen atom. Inter­estingly, however, the distortion of the phenanthrene framework, as measured by either the angle between the mean planes of the terminal rings *A* and *C*, or the C4—C4′—C5′—C5 torsion angle (see Fig. 3 ▸), is the largest for the di­chloro derivative **4** (Table 1 ▸), larger than for the di­bromo and diodo compounds. A combination of both size *and* electronegativity may account for compound **4** showing the largest twist of the phenanthrene system in the series of 4,5-dihalophenathrene compounds.

## Supra­molecular features   

**Figure 4:**
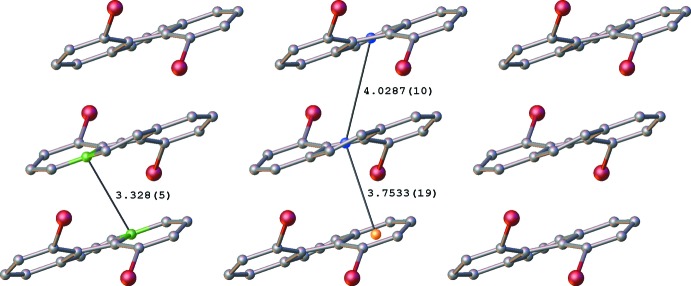
Crystal packing of **2** when viewed along the *b* axis. The separation between the centroids of the middle rings (blue spheres) is slightly longer than that between the centroids of the middle and terminal rings (blue and orange spheres) in adjacent layers. Close contacts are also observed between equivalent carbon atoms in the terminal rings (shown in green) that are offset from each other. All lengths are in Å.

**Figure 5:**
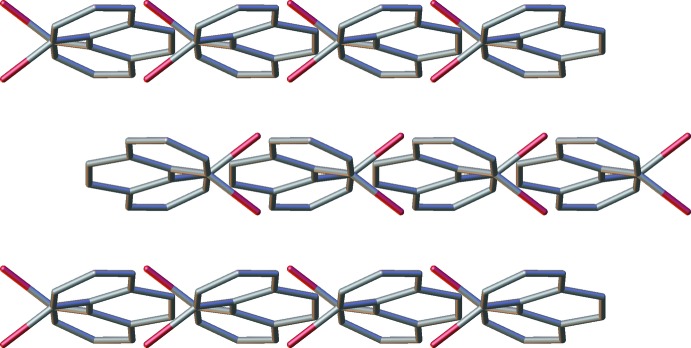
Crystal packing of **2** when viewed along the *a* axis, showing the opposite orientation of mol­ecules in alternating layers.

A view of the crystal packing diagram, along the *b* axis, shows the centroids of the central *B* rings of the phenanthrene units in adjacent layers (marked in blue in Fig. 4 ▸, see Fig. 3 ▸ for ring numbering), separated by a distance of 4.0287 (10) Å. These (blue) centroids are shifted by 2.266 (6) Å relative to each other, indicating a slippage in the stacking arrangement. This ring slippage is also evidenced by the centroid of the *B* ring being at a shorter distance of 3.7533 (19) Å to the *A* ring centroid (shown in orange in Fig. 4 ▸) of the closest phenanthrene unit in an adjacent layer. In addition, short contacts of 3.328 (5) Å are found between C6 (or C6′; refer to Fig. 2 ▸. for atom numbering) and an equivalent carbon atom in an adjacent layer. These atoms, which are in terminal rings offset from each other, are shown in green in Fig. 4 ▸. A view along the *a* axis (Fig. 5 ▸) shows the opposing orientation of the mol­ecules in going from one layer to the next, leading to anti-parallel stacks.

## Database survey   

The Cambridge Structural Database (CSD, Version 5.38, update November 2016; Groom *et al.*, 2016 ▸) reveals entries for 4,5-di­fluoro­phenanthrene (refcode: FIXWOY; Cosmo *et al.*, 1987*a* ▸), 4,5-di­chloro­phenanthrene (refcode: FIXWUE; Cosmo *et al.*,1987*b* ▸), and 4,5-di­iodo­phenanthrene (refcode: PIPRUB; Bock *et al.*,1998 ▸). The title compound, 4,5-di­bromo­phenanthrene (**2**), however, is not in the database.

## Synthesis and crystallization   

The di­aldehyde **1** (108 mg, 0.3 mmol), *p*-toluene­sulfonyl hydrazide (114 mg, 0.6 mmol), and toluene (2 mL) were successively added to a flame-dried flask under argon. The milky white mixture was heated at 333 K and stirred for 10 min. More toluene (14 mL) was added, and the solution was cooled to room temperature. Then, 4 Å mol­ecular sieves (100 mg), KO^*t*^Bu (100 mg, 0.9 mmol), Rh_2_(OAc)_4_ (2 mg, 0.005 mmol), and toluene (14 mL) were added successively. The reaction system was degassed with argon and the resulting solution was stirred at 363 K for 1 h, producing a deep brown–red color after 20 min. The mixture was cooled to room temperature and the crude product was purified by silica gel column chromatography to give **2**, as colorless crystals (24 mg, 0.07 mmol, 23%). m.p. 443–444 K; ^1^H NMR (500 MHz, CDCl_3_) δ 7.40 (*m*, 1H), 7.50 (*m*, 1H), 7.70 (*m*, 1H), 7.80 (*m*, 1H). ^13^C NMR (125 MHz, CDCl_3_) δ 135, 132, 129, 128, 127, 126, 122. LRMS (EI) *m*/*z* 335.9 (*M* ^+^), 255, 176. Crystals suitable for X-ray analysis were grown by the slow diffusion of pentane into a concentrated solution of **2** in di­chloro­methane.

## Refinement   

Crystal data, data collection and structure refinement details are summarized in Table 2 ▸.

## Supplementary Material

Crystal structure: contains datablock(s) global, I. DOI: 10.1107/S2056989017003863/zl2696sup1.cif

Structure factors: contains datablock(s) I. DOI: 10.1107/S2056989017003863/zl2696Isup2.hkl

Supporting information file. DOI: 10.1107/S2056989017003863/zl2696Isup3.cml

CCDC reference: 1532540

Additional supporting information:  crystallographic information; 3D view; checkCIF report

## Figures and Tables

**Table 1:** A comparison of selected structural parameters (Å, °) in a series of known 4,5-dihalo­phenanthrene derivatives Refer to Fig. 3 ▸ for parameters used in this table.

Compound	angle between rings *A* and *C*	*X*⋯*X* distance	C4—C4′—C5′—C5 torsion angle	*X*—C4—C5—*X* torsion angle
**3** (*X* = F)*^*a*^*	16.779	2.381	19.954	43.273
**4** (*X* = Cl)*^*a*^*	32.282	3.097	37.738	69.980
**2** (*X* = Br)*^*b*^*	28.51 (14)*^*b*^*	3.277 (13)^*c*^	32.8 (6)	74.70 (14)
**5** (*X* = I)*^*d*^*	29.451	3.610	33.716	78.611

Notes: (*a*) Cosmo *et al.* (1987*b* ▸); (*b*) this work; (*c*) Cosmo *et al.* 1987(*a*); (*d*) Bock *et al.* (1998 ▸).

**Table 2:** Experimental details

Crystal data	
Chemical formula	C_14_H_8_Br_2_
*M*_r_	336.02
Crystal system, space group	Monoclinic, *C*2/*c*
Temperature (K)	173
*a*, *b*, *c* (Å)	16.840 (3), 8.6112 (16), 8.1418 (15)
β (°)	103.735 (2)
*V* (Å^3^)	1146.9 (4)
*Z*	4
Radiation type	Mo *K*α
μ (mm^−1^)	7.03
Crystal size (mm)	0.25 × 0.11 × 0.07

Data collection	
Diffractometer	Bruker D8 QUEST ECO
Absorption correction	Multi-scan (*SADABS*; Krause *et al.*, 2015 ▸)
*T*_min_, *T*_max_	0.47, 0.64
No. of measured, independent and observed [*I* > 2σ(*I*)] reflections	4708, 1203, 1070
*R*_int_	0.033
(sin θ/λ)_max_ (Å^−1^)	0.634

Refinement	
*R*[*F* ^2^ > 2σ(*F* ^2^)], *wR*(*F* ^2^), *S*	0.030, 0.083, 1.06
No. of reflections	1203
No. of parameters	73
H-atom treatment	H-atom parameters constrained
Δρ_max_, Δρ_min_ (e Å^−3^)	1.01, −0.35

Computer programs: *APEX3* (Bruker, 2016 ▸), *SAINT* (Bruker, 2015 ▸), *SHELXS2013* (Sheldrick, 2008 ▸), *SHELXL2014* (Sheldrick, 2015 ▸), *OLEX2* (Dolomanov *et al.*, 2009 ▸) and *publCIF* (Westrip, 2010 ▸).

## supplementary crystallographic information

### Crystal data

**Table:**

C_14_H_8_Br_2_	*F*(000) = 648
*M**_r_* = 336.02	*D*_x_ = 1.946 Mg m^−^^3^
Monoclinic, *C*2/*c*	Mo *K*α radiation, λ = 0.71073 Å
*a* = 16.840 (3) Å	Cell parameters from 3136 reflections
*b* = 8.6112 (16) Å	θ = 2.5–26.8°
*c* = 8.1418 (15) Å	µ = 7.03 mm^−^^1^
β = 103.735 (2)°	*T* = 173 K
*V* = 1146.9 (4) Å^3^	Block, clear colourless
*Z* = 4	0.25 × 0.11 × 0.07 mm

### Data collection

**Table:**

Bruker D8 QUEST ECO diffractometer	1203 independent reflections
Radiation source: sealed tube, Siemens KFFMO2K-90C	1070 reflections with *I* > 2σ(*I*)
Curved graphite monochromator	*R*_int_ = 0.033
Detector resolution: 8.3660 pixels mm^-1^	θ_max_ = 26.8°, θ_min_ = 2.5°
ω and φ scans	*h* = −21→21
Absorption correction: multi-scan (SADABS; Krause *et al.*, 2015)	*k* = −10→10
*T*_min_ = 0.47, *T*_max_ = 0.64	*l* = −10→10
4708 measured reflections	

### Refinement

**Table:**

Refinement on *F*^2^	0 restraints
Least-squares matrix: full	Hydrogen site location: inferred from neighbouring sites
*R*[*F*^2^ > 2σ(*F*^2^)] = 0.030	H-atom parameters constrained
*wR*(*F*^2^) = 0.083	*w* = 1/[σ^2^(*F*_o_^2^) + (0.0557*P*)^2^ + 0.5337*P*] where *P* = (*F*_o_^2^ + 2*F*_c_^2^)/3
*S* = 1.06	(Δ/σ)_max_ = 0.005
1203 reflections	Δρ_max_ = 1.01 e Å^−^^3^
73 parameters	Δρ_min_ = −0.35 e Å^−^^3^

### Special details

**Table:**

Geometry. All esds (except the esd in the dihedral angle between two l.s. planes) are estimated using the full covariance matrix. The cell esds are taken into account individually in the estimation of esds in distances, angles and torsion angles; correlations between esds in cell parameters are only used when they are defined by crystal symmetry. An approximate (isotropic) treatment of cell esds is used for estimating esds involving l.s. planes.

### Fractional atomic coordinates and isotropic or equivalent isotropic displacement parameters (Å^2^)

**Table:**

	*x*	*y*	*z*	*U*_iso_*/*U*_eq_	
Br1	0.41183 (2)	0.94243 (3)	0.60761 (4)	0.03791 (16)	
C1	0.35716 (18)	0.4938 (4)	0.8748 (4)	0.0403 (7)	
H1	0.34	0.3985	0.914	0.048*	
C2	0.31143 (18)	0.6255 (4)	0.8762 (4)	0.0440 (7)	
H2	0.2652	0.6234	0.9242	0.053*	
C3	0.33332 (16)	0.7628 (4)	0.8065 (4)	0.0384 (6)	
H3	0.2992	0.8517	0.7974	0.046*	
C5	0.45980 (15)	0.6426 (3)	0.7686 (3)	0.0295 (6)	
C4	0.40447 (15)	0.7692 (3)	0.7508 (3)	0.0313 (5)	
C6	0.42910 (17)	0.4984 (3)	0.8160 (3)	0.0332 (6)	
C7	0.46876 (18)	0.3558 (3)	0.7880 (4)	0.0400 (7)	
H7	0.4503	0.2602	0.8239	0.048*	

### Atomic displacement parameters (Å^2^)

**Table:**

	*U*^11^	*U*^22^	*U*^33^	*U*^12^	*U*^13^	*U*^23^
Br1	0.0421 (2)	0.0281 (2)	0.0447 (2)	0.00648 (10)	0.01255 (15)	0.00529 (10)
C1	0.0409 (16)	0.0388 (16)	0.0393 (16)	−0.0115 (14)	0.0055 (12)	0.0039 (13)
C2	0.0334 (14)	0.055 (2)	0.0458 (17)	−0.0090 (14)	0.0138 (12)	−0.0033 (14)
C3	0.0327 (13)	0.0406 (16)	0.0411 (15)	0.0008 (12)	0.0073 (11)	−0.0026 (12)
C5	0.0313 (12)	0.0261 (13)	0.0296 (13)	−0.0020 (10)	0.0041 (10)	−0.0016 (9)
C4	0.0336 (13)	0.0275 (13)	0.0323 (12)	−0.0021 (10)	0.0069 (10)	−0.0008 (10)
C6	0.0361 (14)	0.0290 (14)	0.0309 (13)	−0.0031 (12)	0.0006 (11)	0.0027 (11)
C7	0.0508 (17)	0.0229 (13)	0.0420 (16)	−0.0040 (11)	0.0027 (13)	0.0012 (11)

### Geometric parameters (Å, º)

**Table:**

Br1—C4	1.916 (3)	C3—H3	0.95
C1—C2	1.373 (5)	C5—C4	1.419 (4)
C1—C6	1.405 (4)	C5—C6	1.433 (4)
C1—H1	0.95	C5—C5^i^	1.456 (5)
C2—C3	1.398 (5)	C6—C7	1.441 (4)
C2—H2	0.95	C7—C7^i^	1.341 (6)
C3—C4	1.379 (4)	C7—H7	0.95
			
C2—C1—C6	120.7 (3)	C6—C5—C5^i^	117.96 (17)
C2—C1—H1	119.6	C3—C4—C5	122.5 (3)
C6—C1—H1	119.6	C3—C4—Br1	114.7 (2)
C1—C2—C3	119.5 (3)	C5—C4—Br1	121.6 (2)
C1—C2—H2	120.2	C1—C6—C5	120.8 (3)
C3—C2—H2	120.2	C1—C6—C7	120.0 (3)
C4—C3—C2	120.0 (3)	C5—C6—C7	119.0 (3)
C4—C3—H3	120.0	C7^i^—C7—C6	121.13 (18)
C2—C3—H3	120.0	C7^i^—C7—H7	119.4
C4—C5—C6	115.0 (2)	C6—C7—H7	119.4
C4—C5—C5^i^	126.84 (17)		
			
C6—C1—C2—C3	4.9 (4)	C2—C1—C6—C5	4.9 (4)
C1—C2—C3—C4	−5.9 (4)	C2—C1—C6—C7	−169.4 (3)
C2—C3—C4—C5	−3.1 (4)	C4—C5—C6—C1	−13.0 (4)
C2—C3—C4—Br1	164.5 (2)	C5^i^—C5—C6—C1	171.6 (3)
C6—C5—C4—C3	12.2 (4)	C4—C5—C6—C7	161.3 (2)
C5^i^—C5—C4—C3	−172.8 (3)	C5^i^—C5—C6—C7	−14.1 (4)
C6—C5—C4—Br1	−154.6 (2)	C1—C6—C7—C7^i^	171.4 (3)
C5^i^—C5—C4—Br1	20.4 (4)	C5—C6—C7—C7^i^	−2.9 (5)

Symmetry code: (i) −*x*+1, *y*, −*z*+3/2.

### ?

**Table:**
